# Ferroptosis-related gene signature predicts the prognosis of papillary thyroid carcinoma

**DOI:** 10.1186/s12935-021-02389-7

**Published:** 2021-12-14

**Authors:** Jinyuan Shi, Pu Wu, Lei Sheng, Wei Sun, Hao Zhang

**Affiliations:** 1 Department of Thyroid Surgery, The First Hospital of China Medical University, Shenyang, China; 2 Department of Thyroid Surgery, General Surgery, Qilu Hospital of Shandong University, Jinan, Shandong China

**Keywords:** Papillary thyroid carcinoma, Ferroptosis genes, Gene signature, Prognosis, TCGA

## Abstract

**Background:**

Papillary thyroid carcinoma (PTC) is the most common type of thyroid cancer (TC), accounting for more than 80% of all cases. Ferroptosis is a novel iron-dependent and Reactive oxygen species (ROS) reliant type of cell death which is distinct from the apoptosis, necroptosis and pyroptosis. Considerable studies have demonstrated that ferroptosis is involved in the biological process of various cancers. However, the role of ferroptosis in PTC remains unclear. This study aims at exploring the expression of ferroptosis-related genes (FRG) and their prognostic values in PTC.

**Methods:**

A ferroptosis-related gene signature was constructed using lasso regression analysis through the PTC datasets of the Cancer Genome Atlas (TCGA). Gene Ontology (GO) and Kyoto Encyclopedia of Genes and Genomes (KEGG) enrichment analyses were performed to investigate the bioinformatics functions of significantly different genes (SDG) of ferroptosis. Additionally, the correlations of ferroptosis and immune cells were assessed through the single-sample gene set enrichment analysis (ssGSEA) and CIBERSORT database. Finally, SDG were test in clinical PTC specimens and normal thyroid tissues.

**Results:**

LASSO regression model was utilized to establish a novel FRG signature with 10 genes (ANGPTL7, CDKN2A, DPP4, DRD4, ISCU, PGD, SRXN1, TF, TFRC, TXNRD1) to predicts the prognosis of PTC, and the patients were separated into high-risk and low-risk groups by the risk score. The high-risk group had poorer survival than the low-risk group (*p* < 0.001). Receiver operating characteristic (ROC) curve analysis confirmed the signature's predictive capacity. Multivariate regression analysis identified the prognostic signature-based risk score was an independent prognostic indicator for PTC. The functional roles of the DEGs in the TGCA PTC cohort were explored using GO enrichment and KEGG pathway analyses. Immune related analysis demonstrated that the most types of immune cells and immunological function in the high-risk group were significant different with those in the low-risk group. Quantitative Real-Time Polymerase Chain Reaction (qRT-PCR) verified the SDG have differences in expression between tumor tissue and normal thyroid tissue. In addition, cell experiments were conducted to observe the changes in cell morphology and expression of signature’s genes with the influence of ferroptosis induced by sorafenib.

**Conclusions:**

We identified differently expressed FRG that may involve in PTC. A ferroptosis-related gene signature has significant values in predicting the patients’ prognoses and targeting ferroptosis may be an alternative for PTC’s therapy.

**Supplementary Information:**

The online version contains supplementary material available at 10.1186/s12935-021-02389-7.

## Introduction:

Thyroid cancer (TC) is the most common malignant endocrine tumor and its incidence has steadily increased in many countries over the past several decades [1, 2]. Papillary thyroid carcinoma (PTC) is the most universal type of TC, accounting for more than 80% of all cases, with a high 10-year survival rate over 90% [3, 4]. Although the majority of PTC remain indolent, tumor recurrence and metastasis obstruct the clinical management and survival in certain patients [5].

Ferroptosis describes a novel form of regulated cell death that occurs as a consequence of lethal lipid peroxidation, which is distinct from the apoptosis, necroptosis and pyroptosis [6, 7]. It can be induced by experimental compounds or clinical drugs, such as erastin, ras-selective lethal small molecule 3 (RSL3), or sorafenib, in cancer cells and certain normal cells [8]. It's worth noting that sorafenib was approved by the Food and Drug Administration (FDA) for the treatment of multiple types of advanced cancer, including TC [9–12]. Ferroptosis has been implicated in the pathological cell death and its dysregulation has been associated with carcinogenesis, such as pancreatic cancer, hepatocellular carcinoma, kidney and ovary. Ferroptosis may also have a tumor suppressor function that could be harnessed for cancer therapy [13–16]. In addition, a line of evidence from several researches demonstrate the ferroptotic cells could interact with immune cells, such as CD8 + T cells, by releasing chemotaxis, and then modulating the anticancer immunity [17–21]. Lately, ferroptosis was found to be induced in anaplastic thyroid cancer (ATC) by vitamin C via the inactivation of GPX4, reactive oxygen species (ROS) accumulation and iron sustained lipid peroxidation [22]. Numerous studies have reported that ferroptosis-related genes (FRGs) play a pivotal role in predicting tumor prognosis [23–25], however, the association between FRGs and prognosis in patients with PTC remains to be elucidated.

This study aims at exploring the expression of FRGs and their prognostic value in PTC. Differential expression of FRGs was used to establish a prognostic multigene signature. Additionally, we explored the underlying mechanism of the FRGs signature by a series of analyses to illustrate its prognostic value. Finally, signature genes were tested in clinical PTC specimens and adjacent normal thyroid tissues.

## Materials and methods

### Acquisition of gene expression and clinical data

The mRNA sequencing (RNA-seq) data and corresponding clinical information of 507 PTC patients were downloaded from the TCGA database (https://portal.gdc.cancer.gov/repository). The dataset included 510 PTC tissue samples and 58 adjacent normal tissue samples. The gene expression profiles were normalized using the Perl language (http://www.perl.org/). The corresponding FRGs were downloaded from FerrDb (http://www.zhounan.org/ferrdb/index.html), a web-based consortium that provided a comprehensive and up-to-date database for ferroptosis markers, regulatory molecules and associated diseases [26]. After eliminating duplicate items from the whole genes (driver: 150; suppressor: 109; marker: 123), we identified 259 FRGs (Additional file 1). The clinical information for 507 patients was presented on Table 1.

**Table 1:** Demographic and clinical characteristics of patients in the Cancer Genome Atlas papillary thyroid cancer cohort and validated papillary thyroid cancer cohort

Clinicopathological features	TCGA(N = 507)	Validated Cohort(N = 75)
Age (year)		
Mean (SD)	47.26 (15.78)	44.09 (12.65)
< 55岁	340 (67.06%)	57 (76.00%)
≥ 55岁	167 (32.94%)	18 (24.00%)
Gender, n (%)		
Male	136 (26.82%)	19 (25.33%)
Female	371 (73.18%)	56 (74.67%)
Stage, n (%)		
Stage I	285 (56.21%)	61 (81.33%)
Stage II	52 (10.26%)	8 (10.67%)
Stage III	114 (22.49%)	4 (5.33%)
Stage IV	55 (10.85%)	2 (2.67%)
Unknown	2 (0.39%)	0 (0.00%)
T status, n (%)		
T1	144 (28.40%)	46 (61.33%)
T2	167 (32.94%)	5 (6.67%)
T3	171 (33.73%)	14 (18.67%)
T4	23 (4.54%)	10 (13.33%)
TX	2 (0.39%)	0 (0.00%)
N status, n (%)		
N0	231 (45.56%)	25 (33.33%)
N1	226 (44.58%)	50 (66.67%)
NX	50 (9.86%)	0 (0.00%)
M status, n (%)		
MO	283 (55.82%)	73 (97.33%)
M1	9 (1.78%)	2 (2.67%)
MX	215 (42.41%)	0 (0.00%)

### Prognostic validity of the gene signature

Differentially expressed genes (DEGs) related to ferroptosis in tumor tissues and adjacent normal tissues in the TCGA PTC cohort were described using the ‘limma’ package in R (version 4.0.4; https://www.r-project.org/), with a false-discovery rate (FDR) < 0.05. FRGs related to overall survival (OS) were identified with univariate Cox regression analysis. Ferroptosis-related DEGs were used to construct a protein–protein interaction network with a minimum required interaction score ≥ 0.15 in the STRING database (version 11.0; https://string-db.org/). Then, we also explored their correlations using the ‘igraph’ package in R. The least absolute shrinkage and selection operator (LASSO) Cox regression model was utilized to construct a multigene signature of the ferroptosis-related DEGs in the TCGA cohort by the the ‘glmnet’ package in R. The formula was established as follows: score = e sum (each gene’s expression × corresponding coefficient). The patients were stratified into high-risk and low-risk groups based on the median value of the risk score. The OS and disease-free survival (DFS) differences between high-risk and low-risk groups were compared by the ‘survival’ and ‘survminer’ package in R. Kaplan–Meier curve was implemented to visualize the survival. The receiver operating characteristic (ROC) analysis was used to examine the sensitivity and specificity of survival prediction using the independent risk factors by the ‘timeROC’ package in R. Univariate and multivariate Cox regression analyses were performed to determine the prognostic value of the risk score and various clinical characteristics. Hazard ratios (HRs) and 95% confidence intervals (CIs) were estimated. For the clinical stratified analysis, patients were divided into different subgroups according to the clinicopathologic features, include age, gender, stage and TNM stages. Kaplan–Meier survival analysis was presented by the ‘survival’ and ‘survminer’ package in R between high and low risk score groups in different subgroups.

### Functional enrichment analysis

The "clusterProfiler" R package was utilized to conduct Gene Ontology (GO) and Kyoto Encyclopedia of Genes and Genomes (KEGG) analyses based on the DEGs (|log2FC|≥ 1, FDR < 0.05) between the high-risk and low-risk groups.

### Immune-related analysis

Immune infiltration in PTC was investigated by calculating single-sample gene set enrichment analysis (ssGSEA) scores of 16 immune cells and 13 immune-related pathways using the ‘gsva’ package in R. The correlation between the prognostic signature and immune cells was performed by Pearson correlation analysis via CIBERSORT (http://cibersort.stanford.edu/).

### Statistical analysis

Data were all analyzed using Bioconductor packages in R software. The significance of the differences in the expression of FRGs between tumor and normal tissues was assessed by the Wilcoxon test. Univariate and multivariate cox regression analyses were used to evaluate the correlation between the affecting factors and patients’ OS or DFS. Mann–Whitney test with *P* values adjusted by the Benjamini-Hochberg (BH) method was used to compare the ssGSEA scores of immune cells or pathways between the high-risk and low-risk groups. *P* value < 0.05 was set as statistically significant for all the analyses.

### Quantitative real-time PCR

Quantitative Real-Time PCR (qRT-PCR) was performed on 75 pairs of tumor tissues and adjacent normal tissues to validate the mRNA expression levels of the ten signature genes. Consent form was obtained from each patient involved in this study, and the study approved by the Ethics Committee of the First Hospital of China Medical University (Ethical Approval Number: [2021]82). The clinical information for the 75 patients was presented on Table 1. Total RNA was extracted from the sample tissues via RNAiso Plus (TAKARA, China), followed by reverse transcription into cDNA. qRT-PCR was carried out using the TB Green R Premix Ex TaqTM II kit (TAKARA, China). GAPDH served as an internal control. The relative expression levels were quantified by the Ct (2^−ΔΔCt^) method . The primer sequences were listed (Table 2).

**Table 2:** Primer sequences for qRT-PCR

Primer	Sequence (5′-3′)
ANGPTL7-F	TAGAGATGGAGGACTGGGAGG
ANGPTL7-R	GTGCACACTTGTCCAAGCAG
CDKN2A-F	GGGAGCAGCATGGAGCCG
CDKN2A-R	CTGGATCGGCCTCCGACCGTA
DPP4-F	AGTGGCGTGTTCAAGTGT
DPP4-R	AGTGGCTCATGTGGGTAT
DRD4-F	GGGAAGGACAGTGCTTGGATCTG
DRD4-R	AGTGCTCTAGATAAGTTGGCAAATGCA
ISCU-F	CCAGGTGGATGAAAAGGGGAA
ISCU-R	GCAGAGTTCCTTGGCGATGT
Srxn1-F	CCCACTGGACCAACTTCTGT
Srxn1-R	GTGGCTAGCTCAGACCAAGG
TXNRD1-F	AAATTCTTAGGACGGTCGGG
TXNRD1-R	AGTCTGCCCTCCTGATAAGC
TFRC-F	TCAGTTTCCACCATCTCG
TFRC-R	AAGTCTCCAGCACTCCAA
TF-F	TCAGCAGAGACCACCGAAGACT
TF-R	GACCACACTTGCCCGCTATGTA
PGD-F	GTTCCAAGACACCGATGGCAAAC
PGD-R	CACCGAGCAAAGACAGCTTCTC
GAPDH-F	GTCTCCTCTGACTTCAACAGCG
GAPDH-R	ACCACCCTGTTGCTGTAGCCAA

### Cell culture and reagents

Human thyroid cancer cell lines K1(European Collection of Cell Cultures) and KTC-1(Chinese Academy of Sciences Stem Cell Bank) were cultured in RPMI-1640, comprising 10% fetal bovine serum (FBS), 1% non-essential amino acids(NEAA), 1% Glutamax and 1% Sodium Pyruvate at the humidified chamber of 37 °C and 5% CO2. on. Sorafenib (S7397) was obtained from Selleck( Houston, TX, USA).

### Cell viability assay

Cells (3000 to 4000/well) were seeded in 96-well plates. After a 24 h culture, cells were treated with different doses of sorafenib for 24 h. The Cell Counting Kit-8(CCK‑8; MedChem Express, China) assay was then carried out to assess the effect of sorafenib on cell viability, and IC50 values were calculated according to the instructions.

### Transmission electron microscopy (TEM)

Cell samples were fixed with 2.5% glutaraldehyde, dehydrated in acetone solutions at increasing concentrations, and embedded in an epoxy resin. Then, the Sections (70–90 nm) were stained with lead citrate and uranyl acetate. Ultrastructural images were then captured with a transmission electron microscope (Hitachi H7650, Japan).

## Results

### Identification of 15 differentially expressed prognostic FRGs

**Fig. 1:**
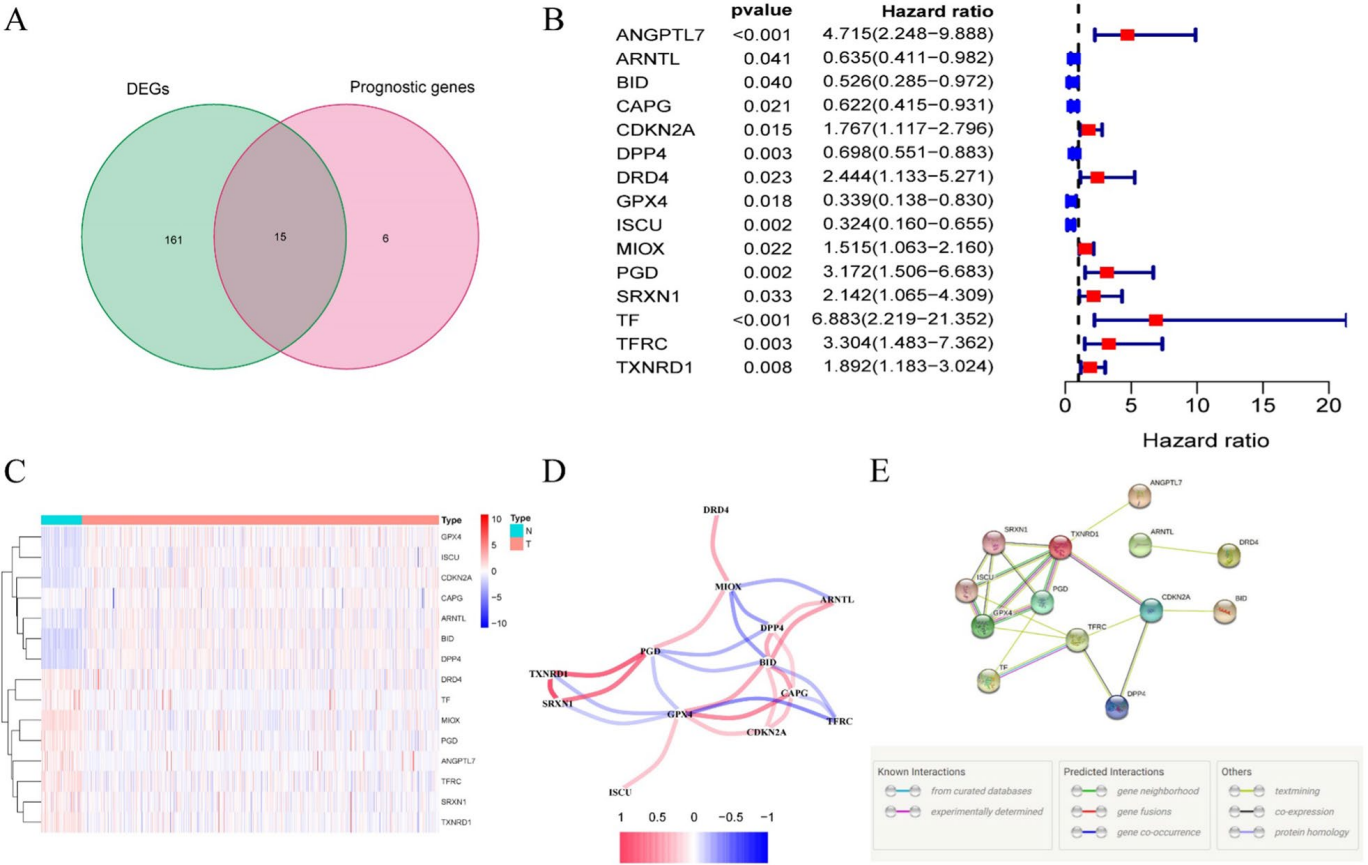
Identification of differentially expressed genes related to ferroptosis in TCGA papillary thyroid cancer cohort. **A** 15 out of 21 prognostic ferroptosis-related genes were differentially expressed between tumor tissues and adjacent normal tissues; **B** univariate Cox proportional regression analysis showed the 15 differentially expressed prognostic ferroptosis-related genes were significantly associated with overall survival; **C** the heatmap of the expression of 15 differentially expressed prognostic ferroptosis-related genes in tumor and normal thyroid. **D** the correlation network of 15 differentially expressed prognostic ferroptosis-related genes. Correlation coefficients are represented by different colors. **E** the PPI network provided interactive information among the 15 differentially expressed prognostic ferroptosis-related genes

Most of the FRGs (176/259, 68.0%) were differentially expressed between tumor and adjacent normal tissue, and 15 of them were correlated with OS in the univariate Cox regression analysis (Fig. 1A, B). The heatmap exhibited the expression difference of 15 intersect genes between tumor tissues and adjacent nontumorous tissues (Fig. 1C). The correlation network and protein–protein interaction network provided interactive information among these differentially expressed prognostic FRGs (Fig. 1D, E).

### Establishment of a 10-FRGs signature predicting the prognosis of PTC

**Fig. 2:**
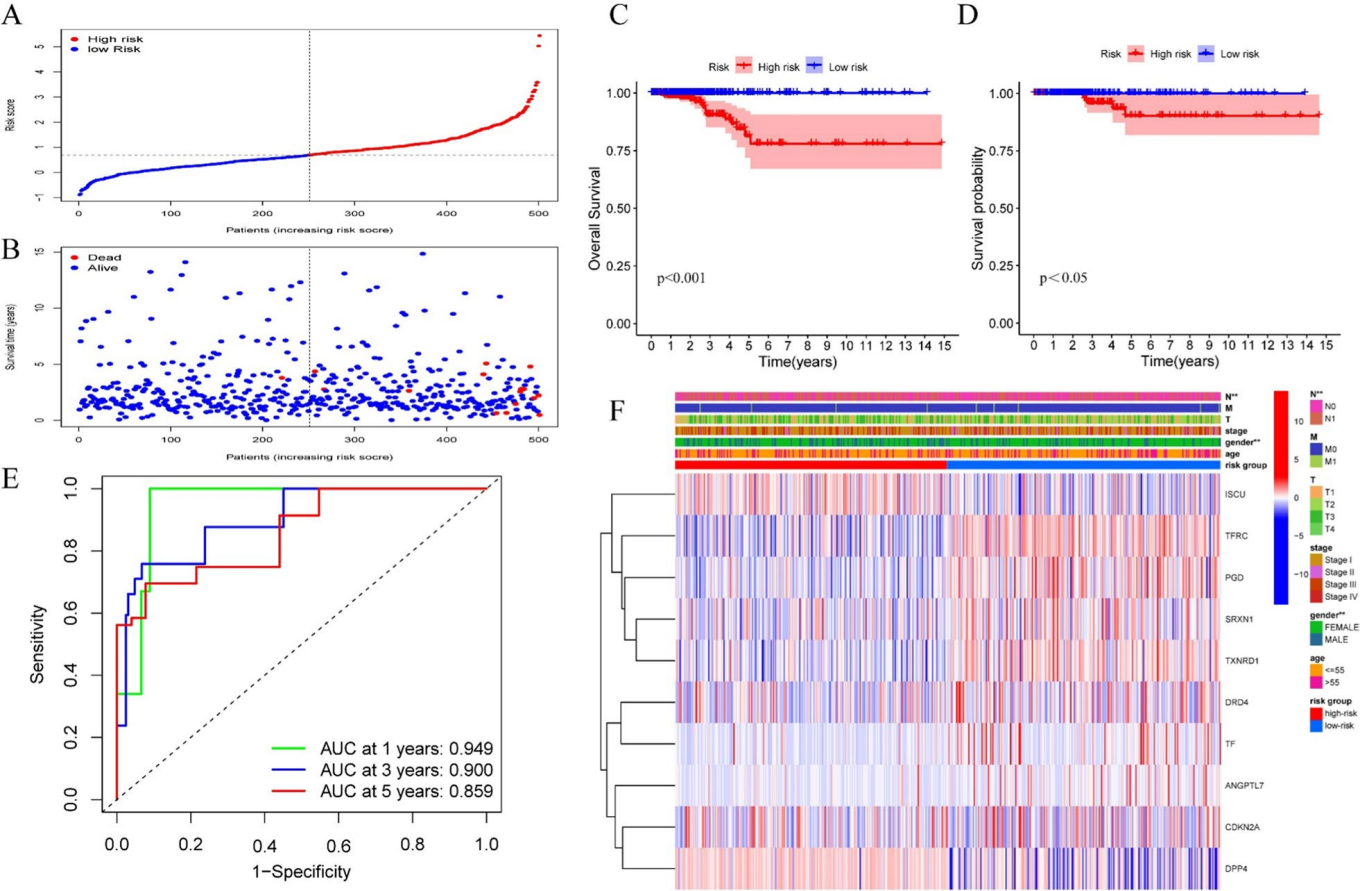
Establishment of a ferroptosis-related gene signature in TCGA papillary thyroid cancer cohort. **A** Signature-based risk scores were used to classify patients as high risk or low risk; **B** survival of high-risk and low-risk patients; **C** Kaplan–Meier curves for overall survival (P < 0.001); **D** Kaplan–Meier curves for disease free survival (P < 0.05); **E** time-dependent receiver operating characteristic curves validated the predictive performance of the signature-based risk score for overall survival; **F** the heatmap for ferroptosis-related genes prognostic signature and clinicopathological manifestations (*P < 0.05, **P < 0.01, ***P < 0.001)

Lasso Cox regression analysis was utilized based on the 15 differentially expressed prognostic FRGs, and a 10-gene signature was constructed. The risk score for each patient was calculated using the following formula: risk score = ANGPTL7 × 1.52040 + CDKN2A × 0.28426 + DPP4 × (− 0.15329) + DRD4 × 0.56101 + ISCU × (− 0.55270) + PGD × 0.31588 + SRXN1 × 0.29977 + TF × 1.38073 + TFRC × 0.60187 + TXNRD1 × 0.15114. Patients were classified as high risk (n = 250) or low risk (n = 251) based on their signature-based risk scores (Fig. 2A, the median value of the risk score = 0.691487). The scatter diagram showed the patients survival status (Fig. 2B). Kaplan–Meier survival curves confirmed that OS and DFS were significantly worse in high-risk compared with low-risk patients (Fig. 2C, D). When the risk score > 1.72, the patient's mean 5-year survival is < 50% (Additional file 2). Time-dependent ROC curves were used to evaluate the predictive performance of the signature-based risk score for OS. The area under the curve values were: 0.949 for 1-year OS, 0.900 for 3-year OS, and 0.859 for 5-year OS, respectively (Fig. 2E). The heatmap indicated the significant differences in gender and N stage between high- and low-risk groups (Fig. 2F).

### The 10-genes signature is an independent prognostic indicator for OS and DFS

**Fig. 3:**
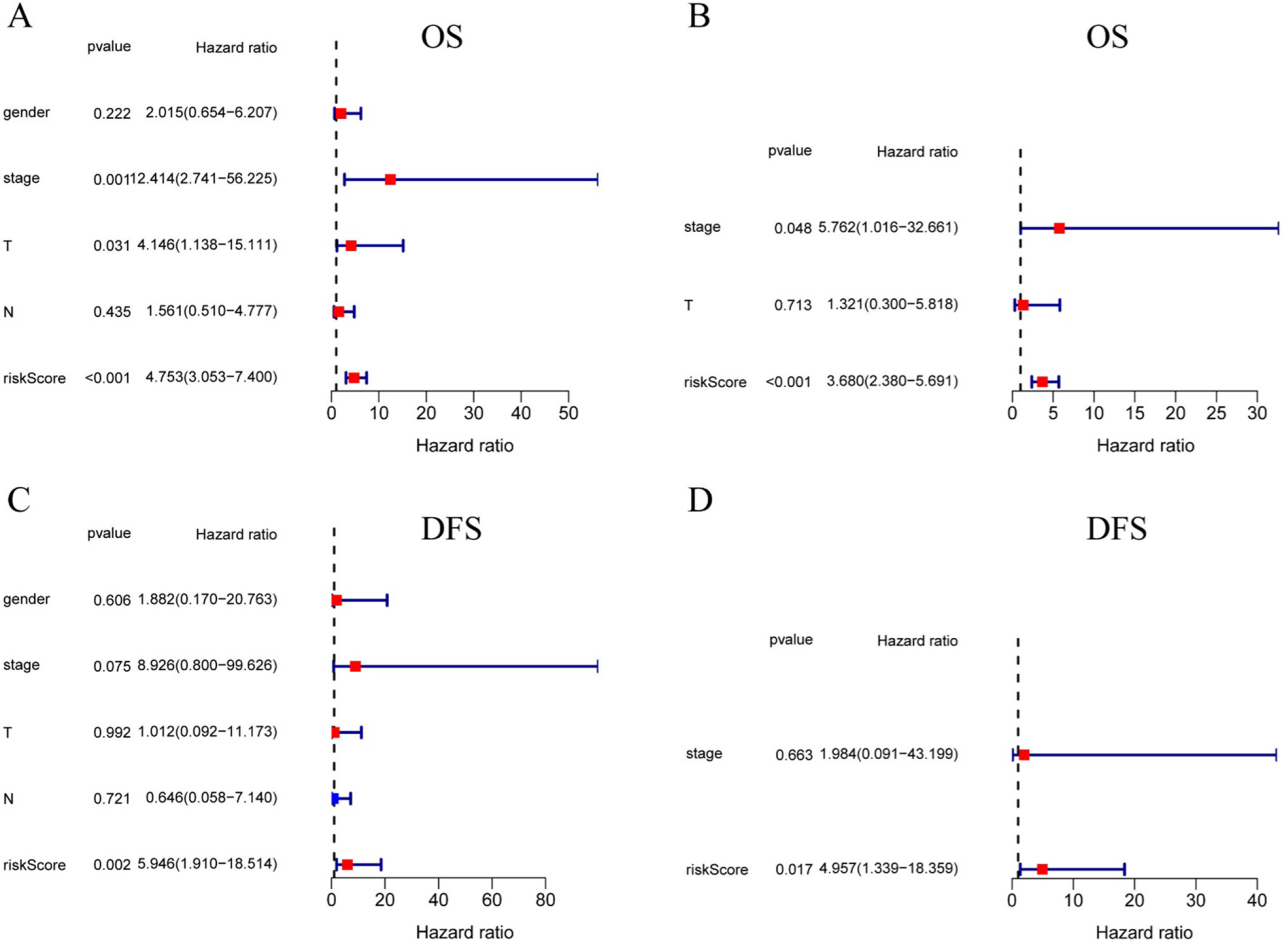
The association between clinicopathological factors and OS or DFS in patients with papillary thyroid cancer. Univariate Cox regression analyses **A** and multivariate Cox regression analyses **B** of the association between clinicopathological factors and OS. Univariate Cox regression analyses **C** and multivariate Cox regression analyses **D** of the association between clinicopathological factors and DFS

Univariate and multivariate Cox regression analyses were carried out among the available variables to come to the conclusion that the risk score was an independent prognostic predictor for OS (Fig. 3A, B; univariate: HR = 4.753, 95% CI = 3.053–7.400, *P* < 0.001; multivariate: HR = 3.680, 95% CI = 2.380– 5.691, *P* < 0.001). Univariate and multivariate Cox regression analyses were carried out to the conclusion that the risk score was significantly associated with DFS (Fig. 3c, D; univariate: HR = 5.946, 95% CI = 1.910–18.514, *P* < 0.01; multivariate: HR = 4.957, 95% CI = 1.339–18.359, *P* < 0.05).

### Clinical stratified analysis verifies the signature’s forecasting performance

**Fig. 4:**
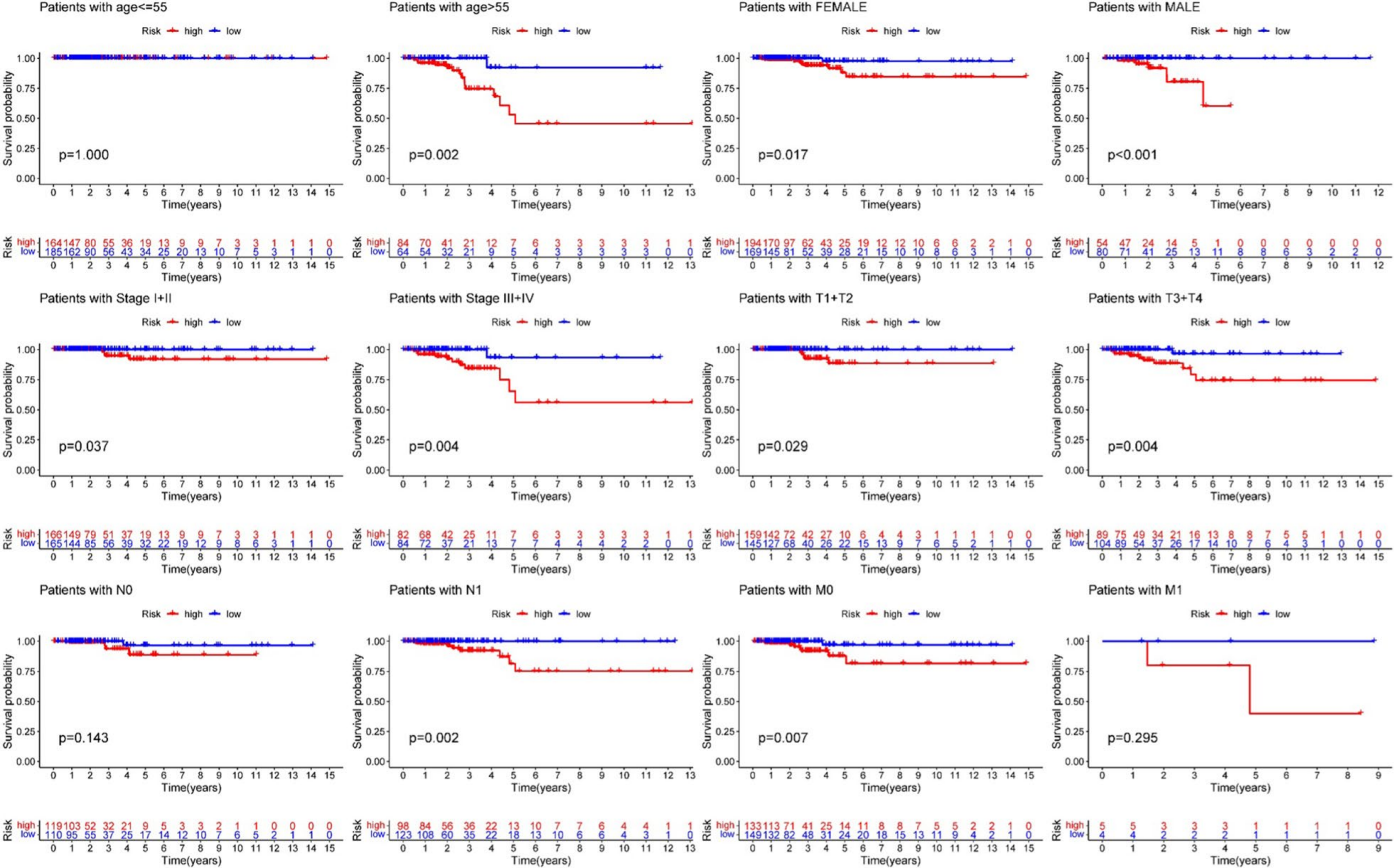
Stratified analysis for the predictive efficacy of the ferroptosis-related gene signature among TCGA papillary thyroid cancer cohort

Clinical stratified analysis was performed to evaluate the forecasting performance of the ten- gene signature (Fig. 4). Although there were no significant difference between high and low risk groups in the OS of three groups (patients with age ≤ 55, N0 and M1), the other 9 clinical groups demonstrated that the high-risk score signified a poorer prognosis in comparison to low-risk score.

### GO and KEGG analyses

**Fig. 5:**
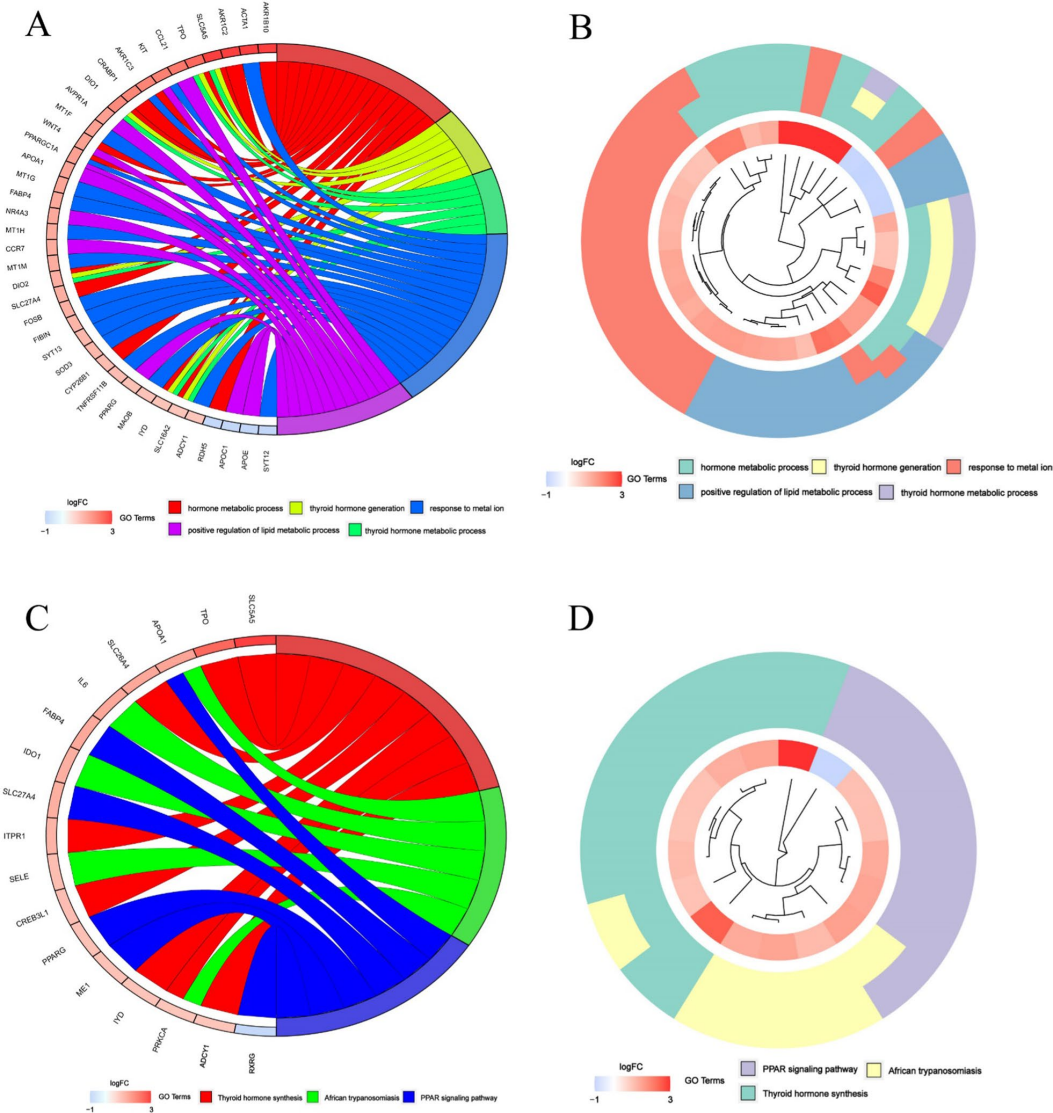
Enrichment analysis in TCGA papillary thyroid cancer cohort. **A**, **B** Gene ontology enrichment. **C**, **D** Kyoto Encyclopedia of Genes and Genomes pathway analyses

The functional roles of the DEGs in high- and low-risk patients in the TGCA cohort were explored using GO enrichment and KEGG pathway analyses. GO analysis showed that the DEGs were mostly enriched in several hormone metabolic processes, especially the thyroid hormone (Fig. 5A, B). Beyond that, enrichment was also observed in the metabolism of lipid and the activation of receptor (*q* value < 0.05). KEGG analysis showed that DEGs were mostly enriched in the thyroid hormone synthesis and immune-related pathways such as the PPAR signaling pathway (Fig. 5C, D; *q* value < 0.05).

### Immune function analysis found that the signature is closely related to the immune cells

**Fig. 6:**
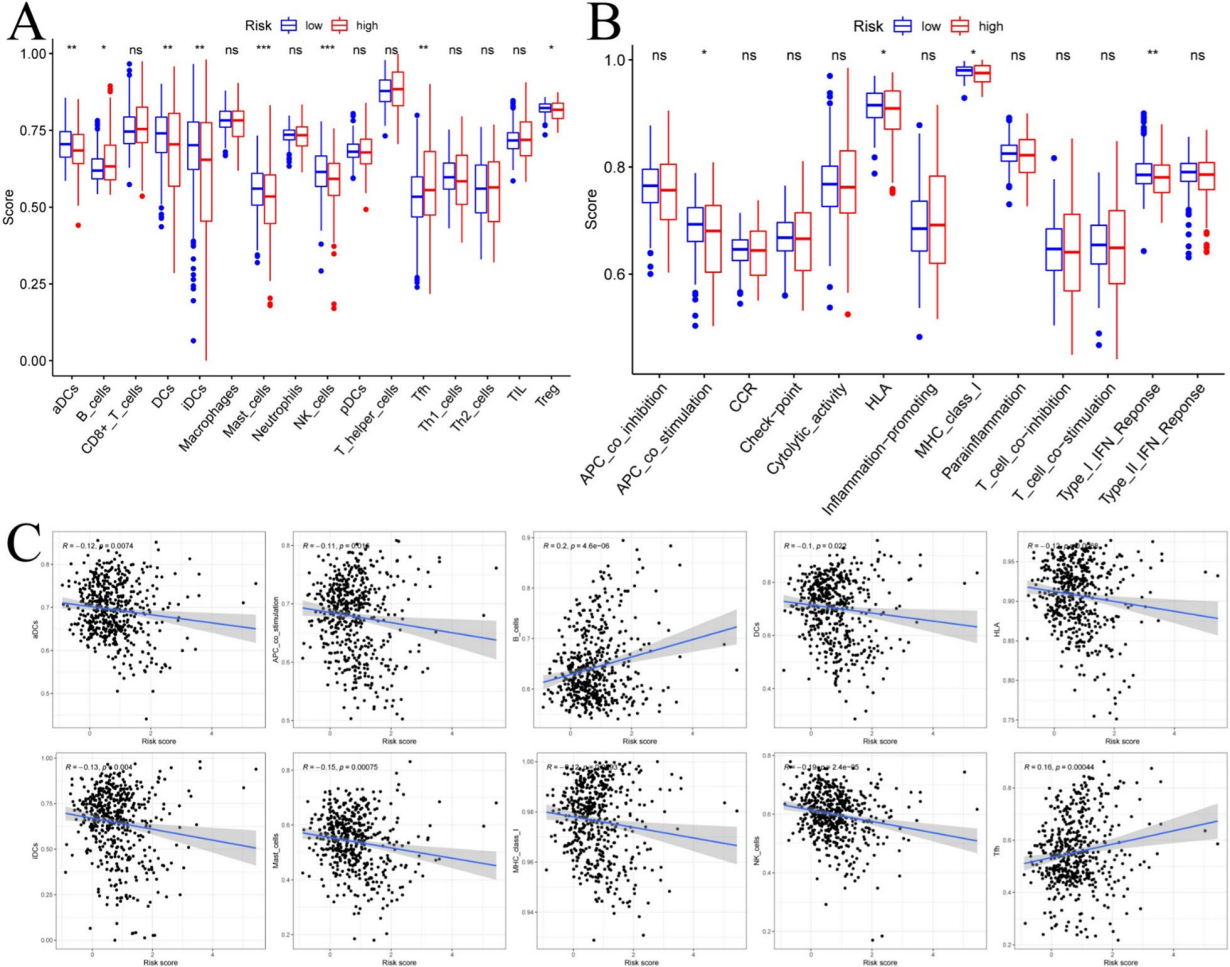
Immune function analysis of ferroptosis-related gene signature in TCGA papillary thyroid cancer cohort. **A**, **B** Comparison of the ssGSEA scores of 13 immune-related functions and 16 immune cells between the high-risk and low-risk groups. **C** Relations between immune cells and the ferroptosis-related gene signature via CIBERSORT. (*ns* not significant; *P < 0.05; **P < 0.01; ***P < 0.001)

To further explore the correlation between the risk score and immune status, we quantified the enrichment scores of diverse immune cell subpopulations, related functions or pathways with ssGSEA. The results showed the accumulation of various immune cells (such as aDCs, B cells, DCs, iDCs, mast cells, NK cells, Tfh cells and Treg cells) with tumor microenvironment (TME) in the high-risk group were significantly different with those in the low-risk group (Fig. 6A). Moreover, the scores of the immune functions, such as the APC co-stimulation, HLA, MHC class I and Type I IFN response were significantly higher in low-risk group, which indicated anti-tumor immunological function was more active in the low-risk group (Fig. 6B).

The CIBERSORT database was used to better understand characteristics of immune cells and FRGs signature (Fig. 6C). The risk score of the 10-gene signature has negative correlations with aDCs (P < 0.01), APC co-stimulation (P < 0.05), DCs (P < 0.05), HLA (P < 0.01), iDCs (P < 0.01), mast cells (P < 0.001), MHC class I (P < 0.01) and NK cells (P < 0.001), while positive correlations with B cell (P < 0.001) and Tfh (P < 0.001).

### Verification of the 10 included genes by clinical specimens and cell experiments

**Fig. 7:**
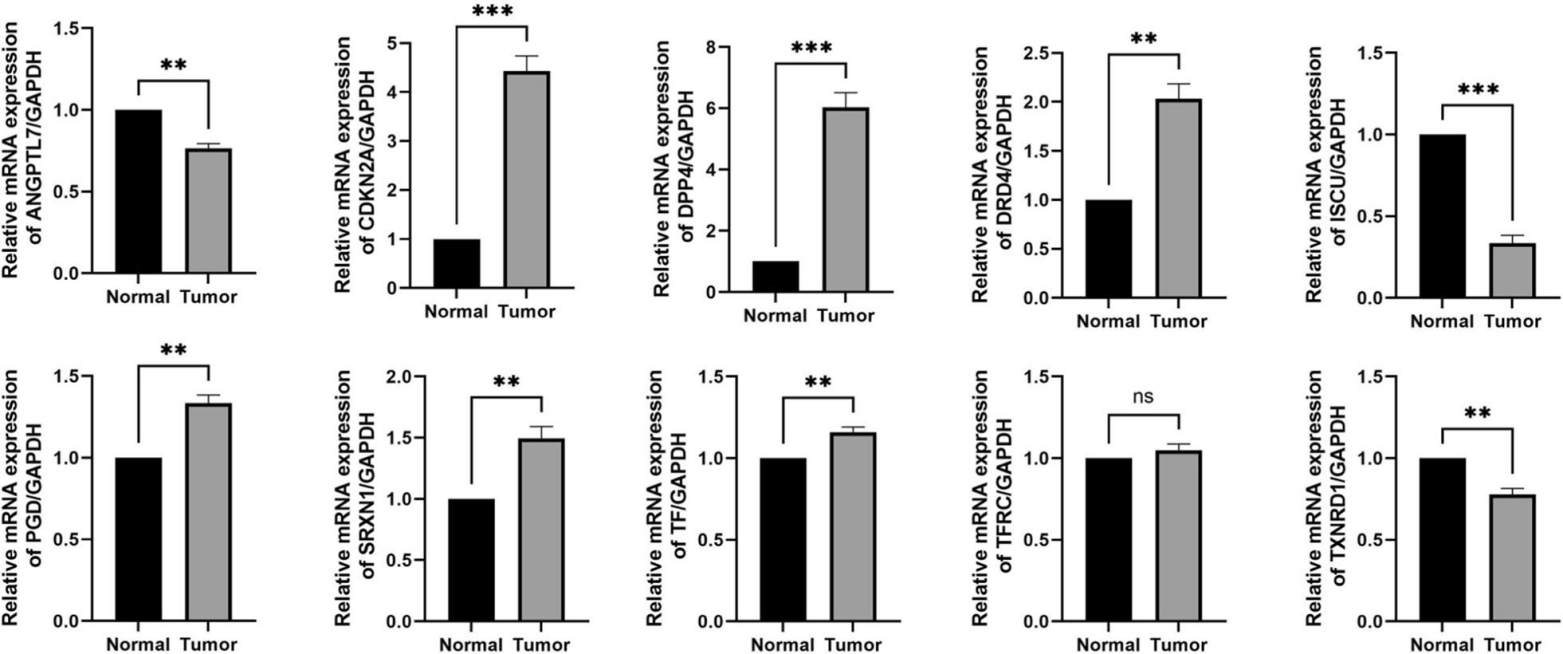
The relative expression levels of the 10 genes in normal and papillary thyroid cancer tissues by qRT-PCR. (*P < 0.05; **P < 0.01, ***P < 0.001, *ns* not significant)

The qRT-PCR was performed on 75 pairs of tumor tissues and adjacent normal tissues to validate the mRNA expression levels of the ten signature genes. The PCR results showed ISCU and TXNRD1 were down-regulated, while the most genes were significantly up-regulated in the PTC tissues. There were no significant differences in the expression of TFRC between the normal and PTC specimens (Fig. 7. *P < 0.05; **P < 0.01, ***P < 0.001, ns: not significant). All results were mainly consistent with the data in TCGA.

**Fig. 8:**
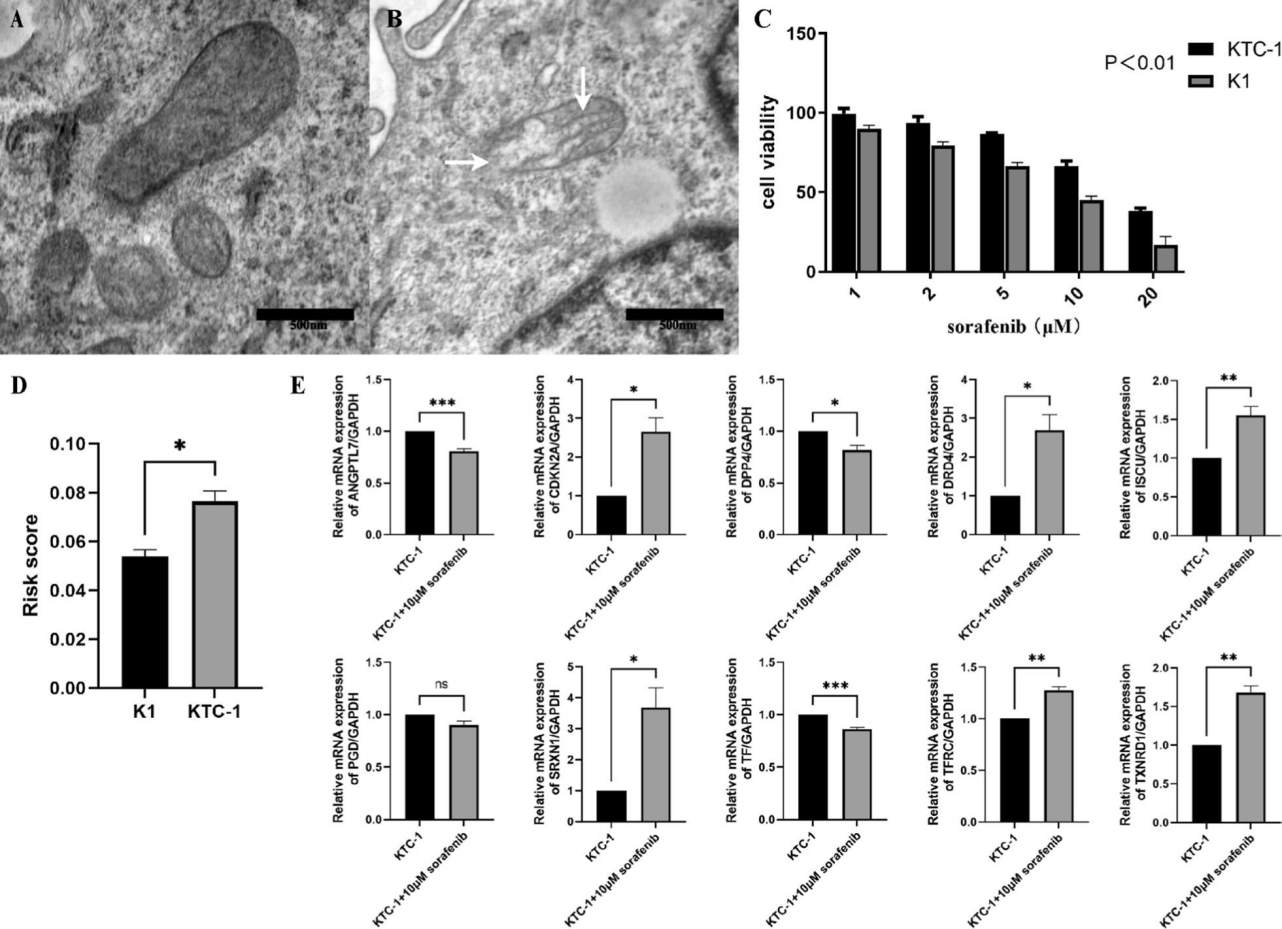
Experiment of thyroid cancer cell line induced by sorafenib. Ultrastructure of the KTC-1 cell line in control group **A** and 10 μM sorafenib for 24 h (**B**). White arrowheads indicate ruptured membrane and the shrunken inner cristae of mitochondria. **C** K1 and KTC-1 cells were treated with different doses of sorafenib for 24 h, and cell growth was measured by Cell Counting Kit-8(P < 0.01). **D** Risk score of K1 and KTC-1(*P < 0.05). **E** The relative expression levels of the 10 genes in KTC-1 and KTC-1 + 10 μM sorafenib for 24 h by qRT-PCR. (*P < 0.05; **P < 0.01, ***P < 0.001, *ns* not significant)

Sorafenib were used in further experiments of thyroid cancer cell lines, as a common inducer of ferroptosis and approved drug for thyroid cancer. TEM showed the ultrastructure of the KTC-1 cell line in control group and 10 μM sorafenib for 24 h. White arrowheads indicate ruptured membrane and the shrunken inner cristae of mitochondria (Fig. 8A, B). K1 and KTC-1 cells were treated with different doses of sorafenib for 24 h, and cell viability was measured by Cell Counting Kit-8. It was found that K1 is more sensitive to sorafenib than KTC-1 (Fig. 8C ,  p<0.01). And we found that the risk score of KTC-1 was higher than K1 (Fig. 8D). The PCR results showed the expression of most genes were significantly changed under the influence of sorafenib. (Fig. 8E, *P < 0.05; **P < 0.01, ***P < 0.001, ns: not significant).

## Discussion

Thyroid cancer is the most common endocrine malignancy with a rapidly increasing incidence worldwide [27]. Although the majority of PTC patients have an excellent prognosis, there are also some patients with recurrence, distant metastasis and even other worse ending, affecting the survival time of patients [28, 29]. Therefore, it is particularly important to carry out risk stratification management to optimize the therapy for PTC patients. Ferroptosis has been proved to participate in tumor proliferation and metastasis, and is closely related to TME and tumor immunity [30, 31]. In this study, we developed a FRGs signature to predict the prognosis of PTC.

From the 15 differentially expressed prognostic FRGs, we established a 10-gene signature. The high expression of ANGPTL7, CDKN2A, DRD4, PGD, SRXN1, TF, TFRC, TXNRD1 represent high risk score and worse prognosis, conversely, the high expression of DPP4 and ISCU were associated with lower risk in PTC. ANGPTL7 is the less characterized member of the Angiopoietin-like (ANGPTL) proteins families, who can stimulate human differentiated endothelial cell’s proliferation, motility and invasiveness [32]. Existing research findings implicated ANGPTL7 was a mediator of metastatic progression and a potential target for interference with cancer metastases [33]. Although CDKN2A/p16 is a known tumor suppressor gene, CDKN2A hypermethylation might be a predictive factor for unfavorable prognosis of portion cancer [34]. According to research findings, high level of DRD4 expression has worse survival than low of DRD4 expression in Glioblastomas [35]. DRD4 might regulate chemosensitivity of cancer via inhibiting ferroptosis [36]. Decreasing PGD phosphorylation through EGFR signaling could dramatically attenuate cancer cells’ proliferation, growth and resistance to ionizing radiation [37]. Beyond that PGD inhibition significantly inhibits growth and survival of ATC cells and sensitizes ATC cells to doxorubicin treatment [38]. SRXN1 is a pivotal regulator of the antioxidant response in eukaryotic cells. SRXN1 could promotes tumor growth and metastasis and correlates with worse prognosis and decreased survival in different carcinoma [39, 40]. TF and TFRC are two key molecules in transportation of iron ion during ferroptosis, and both genes were low expressed in tumor tissues. TF could co-work with thyroid hormones in differentiation and other biological processes, and serum TF may also have a further role in stimulating cell proliferation [41, 42]. TFRC Is a specific ferroptosis marker and may could characterize the extent of ferroptosis [43]. The silencing of TFRC gene reduces liver cancer cell growth and survival [44]. Regulation of TXNRD1 could affect the proliferation, invasion and migration of carcinoma [45, 46]. The inhibition of TrxR1 blocks STAT3 activity and induces cancer cell death [47]. DPP4 has been reported as prognostic marker and therapeutic target of PTC [48]. The activity of DPP4 could down-regulation by TP53 to limits erastin-induced ferroptosis [49]. ISCU, a mitochondrial protein, significantly attenuates ferroptosis by regulating iron metabolism, rescuing the mitochondrial function and increasing the level of GSH [50]. Influenced by TP53, the expression of ISCU is related with the maintenance of iron homeostasis in hepatocellular carcinogenesis [51]. It should be noted that the relationship of ten signature’s genes with ferroptosis and thyroid cancer requires further clarification.

Functional analyses showed that the DEGs in the high-risk and low-risk patients identified in the present study were enriched in PPAR signaling pathway, which is existed as three different isoforms (α, γ, and δ) that play major roles in adipose tissue development and lipid metabolism [52, 53]. PPARs are expressed in immune cells and have an emerging critical role in immune cell differentiation and fate commitment [54]. Moreover, previous studies founded that PPARα activity is essential to regulate lipids through promoting ferroptosis [55]. Presented data suggested that PPARγ may promote growth and invasion of cancer cells and play a detrimental role in thyroid cancer [56, 57]. The metabolic processes of thyroid hormone (TH) have a certain influence on the proliferation and anti-apoptosis of thyroid cancer [58]. The existed research shows that the TH effects the differentiation and microenvironment of various tumors [59, 60]. Targeting TH in the thyroid cancer may improve treatment strategies.

There is a tight crosstalk between ferroptosis and antitumor immunity [61]. Different immune cells and mediators can contribute to thyroid cancer development and progression through various functional pathways [62, 63]. This study found that several kinds of tumor-specific cellular immunity and immune-related functions were altered in high-risk compared with low-risk patients. The previous study found that the accumulation of DCs in PTC was possibly correlated with favorable clinical course [64]. iDC were markedly reduced in poorly differentiated thyroid carcinoma(PDTC) and anaplastic thyroid carcinoma(ATC) [65], which partly supported the differences in iDCs between the high and low risk groups in the signature. The increasing of NK cells in the tumor microenvironment has an inversely correlate with advanced stages in patients with PTC [66]. The suppression of NK cell cytotoxicity and differentiation contributed to immune escape of thyroid cancer [67]. Ferritin heavy chain in tumor cells may modulate the expression of MHC class I molecules and influence NK cells, which may explain the association between ferroptosis and NK activation [68]. It has been reported that the exacerbation of ferroptosis via activating IFN-γ/ASK1/JNK signaling pathway [69] revealed the possible connection between the IFN-γ and ferroptosis. Type II IFN response played a pivotal function on cancer immune surveillance, stimulating antitumor immunity and promoting tumor recognition and elimination [70]. The prognostic signature showed a statistically significant negative correlation with most immune cells, at the same time, the immune score of the high-risk group is just below the low-risk group. Previous research demonstrated that mast cells have a contributing to thyroid carcinoma growth and invasiveness [71]. It was reported that mast cells infiltration was correlated with extrathyroidal extension of tumors and tumor invasiveness [72]. But the low numbers of tryptase mast cells were also associated with poor OS and more advanced stage [73]. The role of mast cells in PTC prognosis warrants further research.

The number of bioinformatics articles about ferroptosis is huge, and there are some similarities inevitably. In our manuscript, the signature was not only related to OS, but also related to DFS of thyroid cancer. So, the signature has better predictive performance. In addition, we stimulated thyroid cancer cell by sorafenib, and verified the expression change of the signature genes.

Although we verified the ten signature genes expression in 75 pairs of tumor tissues and paracancerous tissues, as well as PTC cell lines induced by sorafenib, by qRT-PCR and got a result that almost matched the TCGA, the prognostic predictive value of the ten-gene signature is based on a single cohort. Future studies involving larger independent cohorts should be conducted to validate our findings.

## Conclusion

In conclusion, here we revealed the expression and prognostic value of FRGs in PTC. Additionally, we developed a risk signature that divided PTC patients into high-risk and low-risk groups with significantly different OS and DFS. These results may allow clinicians to determine individualized treatment for PTC patients with different clinical features and provide crucial evidence supporting further research of the role of ferroptosis in PTC.

## Supplementary Information

**Additional file 1.** A list of 259 ferroptosis-related genes identified from FerrDb.

**Additional file 2.** Kaplan–Meier curves for overall survival at the best cut-off value of the risk score.

## Data Availability

Gene expression profiles, clinical information and mutation data of THCA in this study are available from the public database (TCGA, https://portal.gdc.cancer.gov/).
