# Supplementary material for: Ferroptosis-related gene signature predicts the prognosis of papillary thyroid carcinoma
Source: Cancer Cell Int. 2021 Dec 14;21:669. doi: 10.1186/s12935-021-02389-7 (PMC8670268; doi:10.1186/s12935-021-02389-7)
Supplement: Supplementary file 1 — Additional file 1. A list of 259 ferroptosis-related genes identified from FerrDb. [file 12935_2021_2389_MOESM1_ESM.docx]

| ABCC1 | CHAC1 | HIC1 | MTDH | SLC2A12 |
| --- | --- | --- | --- | --- |
| ACO1 | CHMP5 | HIF1A | MTOR | SLC2A14 |
| ACSF2 | CHMP6 | HILPDA | MUC1 | SLC2A3 |
| ACSL3 | CISD1 | HMGB1 | MYB | SLC2A6 |
| ACSL4 | CISD2 | HMOX1 | NCF2 | SLC2A8 |
| ACVR1B | CS | HNF4A | NCOA4 | SLC38A1 |
| AGPAT3 | CXCL2 | HRAS | NF2 | SLC3A2 |
| AIFM2 | CYBB | HSD17B11 | NFE2L2 | SLC40A1 |
| AKR1C1 | DDIT3 | HSF1 | NFS1 | SLC7A11 |
| AKR1C2 | DDIT4 | HSPA5 | NGB | SLC7A5 |
| AKR1C3 | DNAJB6 | HSPB1 | NNMT | SNORA16A |
| ALB | DPP4 | IDH1 | NOS2 | SNX4 |
| ALOX12 | DRD4 | IFNG | NOX1 | SOCS1 |
| ALOX12B | DRD5 | IL33 | NOX3 | SP1 |
| ALOX15 | DUOX1 | IL6 | NOX4 | SQSTM1 |
| ALOX15B | DUOX2 | IREB2 | NOX5 | SRC |
| ALOX5 | DUSP1 | ISCU | NQO1 | SRXN1 |
| ALOXE3 | EGFR | JDP2 | NRAS | STAT3 |
| ANGPTL7 | EGLN2 | JUN | OTUB1 | STEAP3 |
| ANO6 | EIF2AK4 | KEAP1 | OXSR1 | STMN1 |
| ARNTL | EIF2S1 | KIM-1 | PANX1 | TAZ |
| ARRDC3 | ELAVL1 | KLHL24 | PCK2 | TF |
| ASNS | EMC2 | KRAS | PEBP1 | TFAP2C |
| ATF3 | ENPP2 | LAMP2 | PGD | TFR2 |
| ATF4 | EPAS1 | LINC00336 | PHKG2 | TFRC |
| ATG13 | FADS2 | LINC00472 | PIK3CA | TGFBR1 |
| ATG16L1 | FANCD2 | LOC284561 | PLIN2 | TLR4 |
| ATG3 | FBXW7 | LOC390705 | PLIN4 | TMBIM4 |
| ATG4D | Fer1HCH | LONP1 | PML | TNFAIP3 |
| ATG5 | FH | LPCAT3 | PRDX1 | TP53 |
| ATG7 | FLT3 | LPIN1 | PRDX6 | TP63 |
| ATM | FTH1 | LURAP1L | PRKAA1 | TRIB3 |
| ATP5MC3 | FTL | MAFG | PRKAA2 | TSC22D3 |
| ATP6V1G2 | FTMT | MAP1LC3A | PROM2 | TUBE1 |
| AURKA | G6PD | MAP3K5 | PSAT1 | TXNIP |
| BACH1 | G6PDX | MAPK1 | PTGS2 | TXNRD1 |
| BAP1 | GABARAPL1 | MAPK14 | RB1 | UBC |
| BECN1 | GABARAPL2 | MAPK3 | RELA | ULK1 |
| BID | GABPB1 | MAPK8 | RGS4 | ULK2 |
| BLOC1S5-TXNDC5 | GCH1 | MAPK9 | RIPK1 | VDAC2 |
| BNIP3 | GCLC | MIOX | RPL8 | VEGFA |
| BRD4 | GDF15 | MIR137 | RRM2 | VLDLR |
| CA9 | GLS2 | MIR17 | SAT1 | WIPI1 |
| CAPG | GLUT13 | MIR212 | SCD | WIPI2 |
| CARS1 | GOT1 | MIR30B | SCP2 | XBP1 |
| CAV1 | GPT2 | MIR4715 | SELENOS | YWHAE |
| CBS | GPX2 | MIR6852 | SESN2 | YY1AP1 |
| CD44 | GPX4 | MIR9-1 | SETD1B | ZEB1 |
| CDKN1A | HAMP | MIR9-2 | SIRT1 | ZFP36 |
| CDKN2A | HBA1 | MIR9-3 | SLC1A4 | ZFP69B |
| CDO1 | HELLS | MT1G | SLC1A5 | ZNF419 |
| CEBPG | HERPUD1 | MT3 | SLC2A1 |  |

**Additional file 1:** A list of 259 ferroptosis-related genes identified from FerrDb.
