# Supplementary material for: Ferroptosis-related gene signature predicts the prognosis of papillary thyroid carcinoma
Source: Cancer Cell Int. 2021 Dec 14;21:669. doi: 10.1186/s12935-021-02389-7 (PMC8670268; doi:10.1186/s12935-021-02389-7)
Supplement: Supplementary file 2 — Additional file 2. Kaplan–Meier curves for overall survival at the best cut-off value of the risk score. [file 12935_2021_2389_MOESM2_ESM.docx]

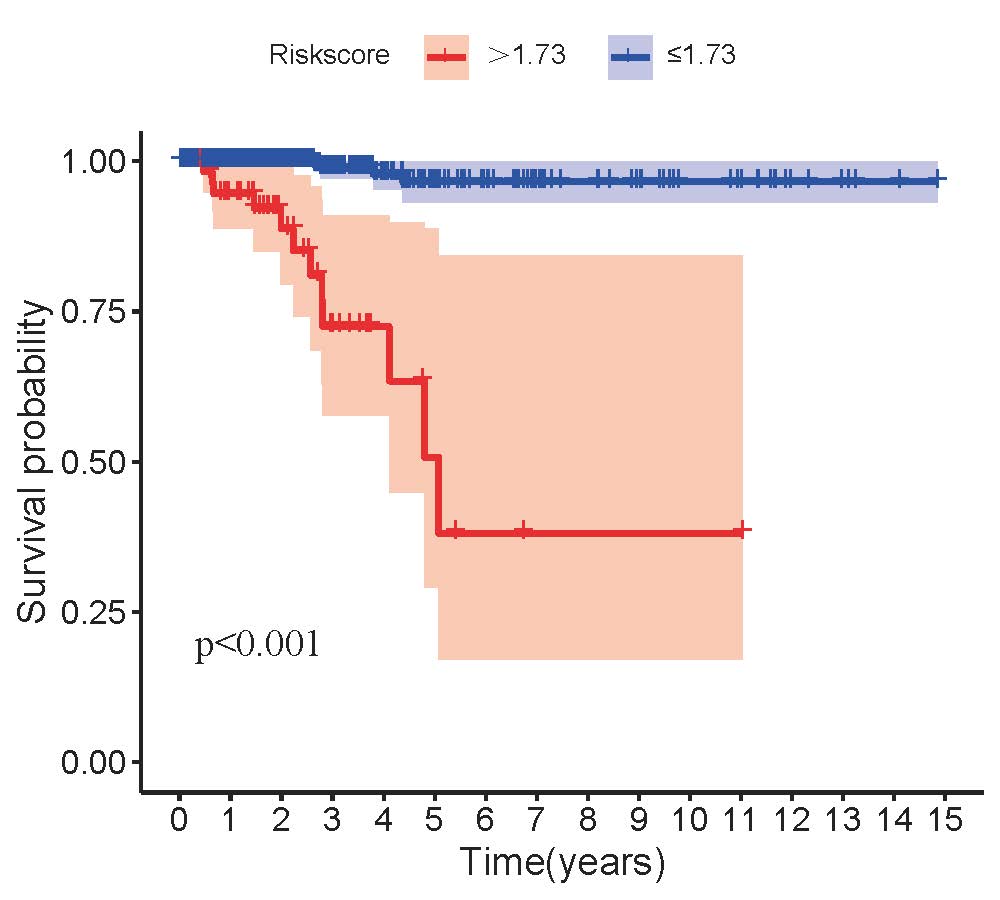


**Additional file 2:** Kaplan–Meier curves for overall survival at the best cut-off value of the risk score.
